# Seasonal and urban effects on the endocrinology of a wild passerine

**DOI:** 10.1002/ece3.1820

**Published:** 2015-11-19

**Authors:** Anja Russ, Susanne Reitemeier, Anne Weissmann, Jutta Gottschalk, Almuth Einspanier, Reinhard Klenke

**Affiliations:** ^1^ Department of Conservation BiologyHelmholtz‐Centre for Environmental Research ‐ UFZPermoserstraße 1504318LeipzigGermany; ^2^ Institute of Physiological ChemistryUniversity of LeipzigAn den Tierkliniken 104103LeipzigGermany

**Keywords:** Circannual rhythmicity, endocrine disruption, light loggers, light pollution, timing of reproduction, urbanization

## Abstract

In order to maximize their fitness, organisms in seasonal environments rely on external cues to optimally time their life‐history stages. One of the most important zeitgeber to time reproduction is the photoperiod, but further environmental cues are assessed to fine‐tune reproduction due to year‐to‐year variation in environmental conditions. However, in urbanized environments, the pervasive artificial light at night has altered the natural signal of light and darkness. Accordingly, artificial light at night was repeatedly shown to affect avian reproductive physiology and to advance seasonal reproduction in birds. However, these experiments were mainly conducted in the absence of further environmental cues to facilitate the investigation of the mechanisms which are still poorly understood. Here, we investigate whether the endocrine system of free‐ranging European blackbirds (*Turdus merula*) correlates with the amount of artificial light at night along a rural to urban gradient while the birds still encounter complementary environmental cues including seasonal variation in day length and temperature. Testosterone and estrone were assessed as metabolites in fecal samples and corticosterone in blood from mist‐netted blackbirds. We demonstrate that seasonal fluctuations in abiotic factors, individual conditions, but also light at night affect the reproductive and stress physiology of wild European blackbirds. Elevated artificial night light intensities were significantly positively correlated with corticosterone and negatively with female estrone levels. No effects of artificial light were found for testosterone levels. Our results suggest that female blackbirds in particular perceive even low levels of artificial light at night as a weak but chronic stressor that interacts with the hypothalamic‐pituitary‐gonadal axis and leads to a reduced secretion of reproductive hormones. These findings point out that the impacts of light pollution are diverse and we only slowly disentangle its multiple effects on physiology, ecology, and biodiversity.

## Introduction

In seasonal environments, organisms need to optimally time life‐history stages to maximize their fitness (Bradshaw and Holzapfel 2007). As most stages require preparations well in advance, proximate environmental cues are assessed to anticipate appropriate conditions in the future, while ultimate factors ensure further fine‐tuning according to year‐to‐year variations (Bradshaw and Holzapfel 2007; Ball and Ketterson 2008). The most important proximate cue is the photoperiod because it is easily assessed and highly reliable over evolutionary time scales (Bradshaw and Holzapfel 2007). In nontropical birds, for example, gonads are usually inactive and considerably regressed in size during the nonbreeding season (Dawson et al. 2001). The increasing photoperiod triggers a hormonal cascade via the hypothalamic–pituitary–gonadal (HPG) axis which stimulates the maturing of the reproductive organs (Dawson et al. 2001). Consequently, the gonads release sex steroids such as testosterone (T) and estrogens which initiate secondary sexual characteristics including a wide range of reproductive behaviors such as territory establishment, courtship display, and nest building (Ball 1993). Toward the end of the breeding season, long photoperiods no longer sustain reproduction, but induce photorefractoriness and birds rapidly regress their gonads (Dawson et al. 2001; Bradshaw and Holzapfel 2007). To break photorefractoriness and close the annual cycle, birds need to experience short days like in late autumn and early winter (Dawson et al. 2001; Bradshaw and Holzapfel 2007). Hence, avian reproduction is closely linked to the seasonal alternation of short and long days.

However, since the dawn of industrialization, an increasing amount of artificial light at night (LAN) scatters through the atmosphere and blurs the natural light signal. In developed areas, not only artificial lighting of roads, office and public buildings, but also private homes cause night skies to exceed the brightness of full‐moon nights (Cinzano et al. 2001; Kyba et al. 2011). Consequentially, the natural darkness decreases to a persistent twilight and the formerly reliable signal of day length loses its accurateness. This loss of the strong day/night signal affects organisms of many different taxa, including humans, due to the widespread interaction between the natural cycle of light and darkness and biological processes (Navara and Nelson 2007). Serious consequences range from disruption of behavior and physiology to changes in species interactions and community structures (Longcore and Rich 2004).

Urban settlements are the areas most affected by LAN and challenge organisms further by, for example, novel food resources, different microclimate, and anthropogenic noise (Partecke et al. 2005; Slabbekoorn and Ripmeester 2008). In particular, the latter seems to be challenging for urban birds as the noise interferes with avian acoustic communication, has the potential to change species composition, and may cause serious fitness consequences (Francis et al. 2009; González‐Oreja et al. 2012; Schroeder et al. 2012). Nevertheless, some species manage to settle and survive in these disturbed habitats. To cope with the new environmental conditions, urban organisms seem to adjust behavioral and physiological traits, as well as life‐history strategies. Becoming tamer, breeding in higher densities, extending the breeding period and daily activity patterns were considered as adaptations to urban life (e.g., Luniak and Mulsow 1988; Bergen and Abs 1997; Partecke et al. 2005; Fuller et al. 2007; Chamberlain et al. 2009; Atwell et al. 2012). These responses to the urban environment are mediated by the organism's endocrine system (Bonier 2012). For example, elevated baseline corticosterone (CORT) as well as reduced CORT response to handling in urban birds were seen as an adaptation to cope with the greater variety of challenges in the urban environment (Bonier et al. 2007; Zhang et al. 2011; Atwell et al. 2012). However, changes in endocrine patterns due to urban influences in general were not consistent between species, sexes, and life‐history stages (Bonier 2012).

A well‐studied example for urban–rural divergence is the European blackbird (*Turdus merula*). Laboratory experiments indicated that LAN has the potential to advance both seasonal reproduction and the onset of morning song activity of the blackbirds (Dominoni et al. 2013a,b). However, in field studies of blackbirds, these patterns were also attributed to better food supply, higher temperature and, regarding the onset of dawn song, also to urban noise (Cuthill and Macdonald 1990; Partecke et al. 2005; Nordt and Klenke 2013).

Despite the increasing amount of research examining the effects of LAN, it is still poorly understood how LAN interacts with the avian endocrinology under natural conditions. Here, we aim to find out whether variations in the release of reproductive hormones in free‐living European blackbirds correlate with LAN while the birds encounter environmental fluctuations in day length and temperature which affect the physiology of the birds in a complementary fashion. We investigated noninvasively the concentrations of T as fecal conjugate, and oestrogen as its main fecal metabolite estrone sulfate (E_1_S), to assess variations in these two sex steroids in the context of environmental, individual, and anthropogenic influences. Although we expected testosterone to show greater variations in males and, thus, be closer linked to male responses to environmental influences whereas E_1_S might represent female responses, both reproductive hormones were investigated in either sex of the blackbirds. A recent study indicated a positive correlation between LAN and baseline concentrations of the main avian glucocorticoid CORT in wild birds (Ouyang et al. 2015). Therefore, we also linked the reproductive hormones to the stress physiology by including plasma concentrations of CORT into the analysis.

## Material and Methods

### Study site and field techniques

**Figure 1:**
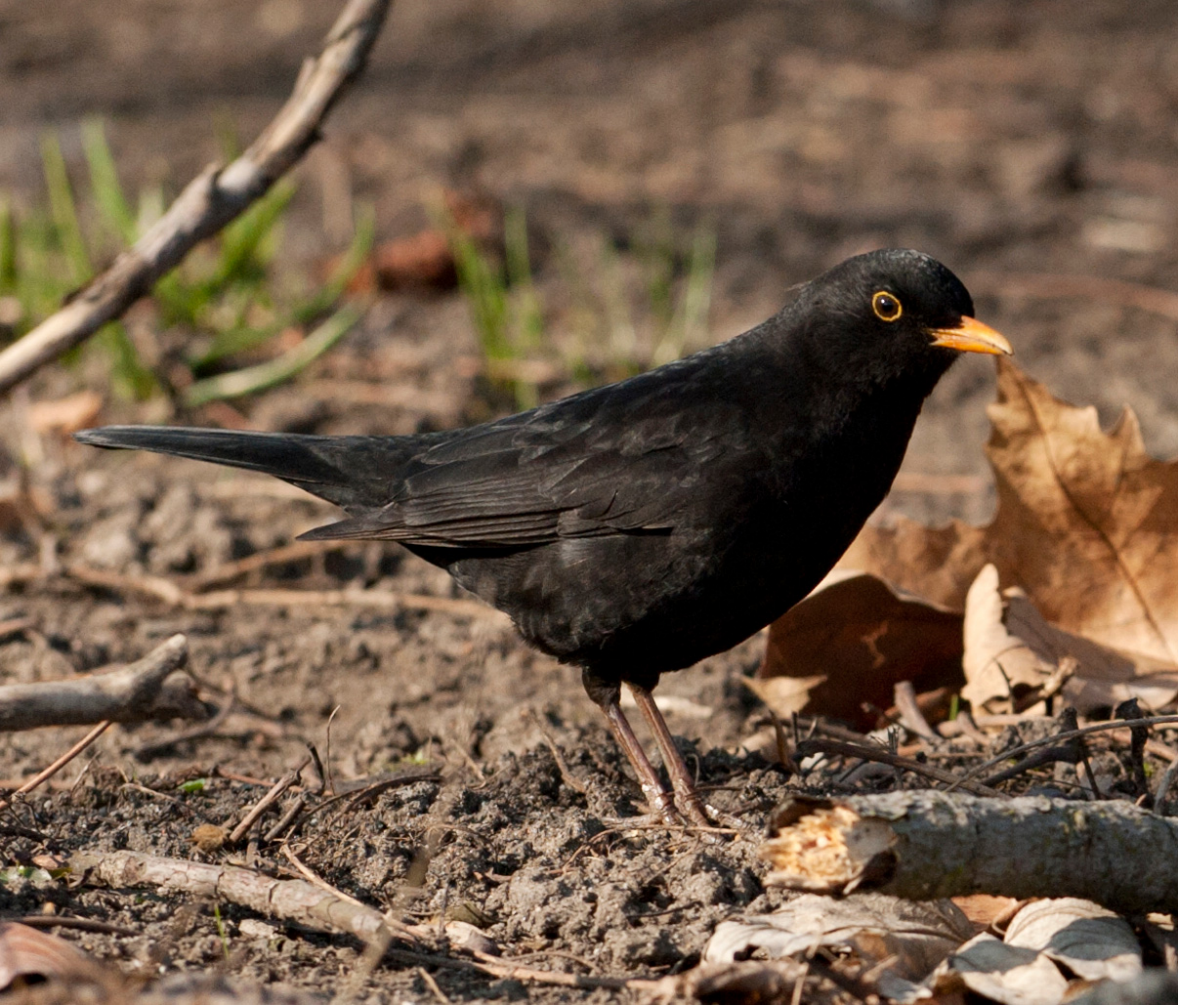
Male European blackbird during the early breeding season. Photograph by A. Künzelmann, UFZ.

**Figure 2:**
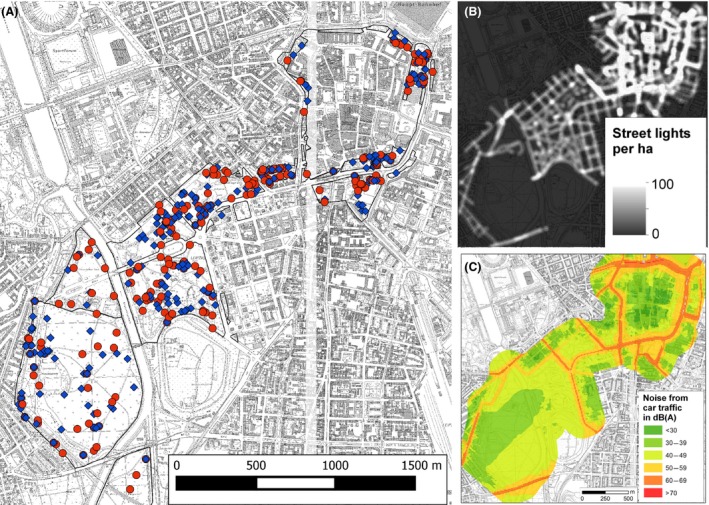
Overview of study area. Sampling of male (blue diamonds) and female (red circles) European blackbirds occurred along a rural to urban gradient (A) ranging from an urban forest in the southwest, across several larger parks to the city center in the northeast with adjacent tiny parks and green spaces. This gradient is also found in the distribution of artificial light (B) and anthropogenic noise from car traffic (C).

Between April 2011 and May 2013, free‐ranging adult blackbirds (Fig. 1) were mist‐netted along a rural–urban gradient in the city of Leipzig, Germany (51°20′ 23″ N, 12°22′ 23″ E). This gradient ranged from an urban forest across several parks to the city center which is confined by a busy and brightly illuminated ring road (Fig. 2). For a detailed description of the study area, see Nordt and Klenke (2013). Captured individuals were banded with a metal ring and an alphanumeric coded color ring for later identification in the field. Birds were weighed to the nearest 0.1 g (TCB 200‐1; Kern, Balingen‐Frommern, Germany), and the tarsus was measured to the nearest 0.01 mm. To determine plasma CORT levels, blood samples (*n *=* *168) were taken by puncturing the brachial vein with a 25‐g needle (Terumo, Eschborn, Germany) and collecting 200–500 *μ*L of blood in a heparinized tube. The time between capture and completed blood sampling was taken for inclusion into further analysis. After bleeding was staunched, the bird was put into an opaque box for approximately 10–15 min to defecate and was released afterward. The box was lined with plastic foil to facilitate the collection of the fecal sample (*n *=* *192) which consisted of liquid and solid components of the droppings and was further referred to as feces. Additional 447 fecal samples were obtained by observing ringed and naive adult blackbirds during activities at the ground until they defecated. Only those droppings with unambiguous identity were collected. Within 1–3 h of collection fecal samples were stored at −20°C. Blood samples were centrifuged (10 min, 500 g) to separate plasma from red blood cells and also stored at −20°C until further processing.

### T and E_1_S hormone assays

Approximately one‐third of sampled feces were discarded because occasionally both methods of fecal sampling did not yield enough material, leaving 389 and 406 samples for the analyses of T and E_1_S, respectively. Of these samples, 54 % originated from male blackbirds and 46 % of females. Ninety‐three T samples (23.4 %) resulted from repeated sampling of 35 birds and 101 E_1_S samples (24.9 %) of 39 birds.

Preconditioning of feces was conducted as previously described elsewhere (Hahn et al. 2011). After lyophilization, the fecal samples were incubated with an enzyme solution (glucuronidase/arylsulfatase in citrate buffer, pH 4.8) to cleave conjugated steroids and thus to detect the total concentration of T and E_1_S. The extraction with diethylether was followed by vaporization of the supernatant and resuspension of the extracts in assay buffer (pH 7.2). The analysis of T and E_1_S was performed via specifically developed competitive enzyme immunoassays (Hahn et al. 2011; Weissmann et al. 2013). Ninety‐six well plates coated with sheep anti‐rabbit IgG and hormone‐specific antibodies (T: in‐house immunization; E_1_S: R522‐2, Clinical Endocrinology Laboratory, UC Davis, USA) as well as hormone‐enzyme conjugates (T‐HRP: Fitzgerald Industries International, North Acton, MA; E_1_S‐HRP: Clinical Endocrinology Laboratory) were used. The calibration curves ranged from 0.05 to 12.5 ng/mL (T) and 0.1 to 10 ng/mL (E_1_S), respectively. Sensitivities were 0.03 ng/mL (T) and 0.04 ng/mL (E_1_S), and recovery rates amounted to 78 % and 95 %, respectively. Interassay/intra‐assay variations were as follows: 4.4%/9.8% (T) and 8.5%/9.4% (E_1_S).

### CORT hormone assay

Of the 168 blood samples, 52% were collected from males and 48% from female blackbirds. Thirty‐five of the samples (20.8 %) originated from repeated sampling of 17 recaptured individuals.

The plasma levels of CORT were quantified by a ^3^H‐radioimmunoassay (RIA) as previously described (Hahn et al. 2011). For sample preparation, disruptive proteins were eliminated by precipitation with ethanol. The supernatant solution was evaporated and resuspended in phosphate buffer. For the RIA, a hormone‐specific antibody (in‐house immunization), unmarked CORT (Serva Electrophoresis GmbH, Heidelberg, Germany) as a standard and [1,2,6,7‐^3^H]‐CORT (Perkin Elmer LAS GmbH, Rodgau Jügesheim, Germany) as a tracer were used. The hormone concentrations of the samples were recalculated by means of a calibration curve (0.1–5.0 ng/mL). The sensitivity of the assay amounted to 0.1 ng/mL. Intra‐assay and interassay coefficients of variation were 6.1 and 8.3%, respectively.

### Environmental parameters

A detailed description of how LAN and anthropogenic noise was quantified can be found in Nordt and Klenke (2013). In brief, data of municipal street lighting were used as a proxy to quantify LAN at the mist‐netting and fecal sampling sites. The municipal traffic and works service, Leipzig, provided the light point data from which a *lamp density* index was calculated using kernel density estimation with a search radius of 50 m and a resolution of 10 m (ArcGIS 10; ESRI, Redlands, CA, USA). Validation of the lamp density index with illuminance measurements at 100 randomly selected points revealed a significant correlation. Thus, the lamp density index was regarded as an accurate indicator of LAN. The corresponding illuminance values (in lux) were calculated by means of this correlation and will be provided to facilitate comparability with other studies.

Traffic noise as another urban factor was considered to interfere with intraspecific acoustic communication and, thus, to stress the birds. Night‐time noise indices of car traffic and tramway were provided by the Environmental Protection Office Leipzig. They are based on separate counts of car and tramway traffic between 10 pm and 6 am, and provide standardized values of anthropogenic noise in dB(A) at a resolution of 10 by 10 m (Bundesministerium der Justiz 2006). Night‐time noise indices were included in the analyses because they overlap with periods of extensive blackbird dawn singing and also with the birds' exposure to LAN. Furthermore, night‐time noise indices are linearly correlated to daytime noise indices at 1000 random points (Pearson *r*_car_ = 0.992, *P* < 0.001, *r*_tramway_ = 0.996, *P* < 0.001) and can thus be used interchangeably.

Both *noise* and *lamp density* values were related to the mist‐netting and fecal sampling sites. Given the relatively coarse spatial resolution of noise and light indices and that resident birds were trapped and sampled at the nest site when rearing chicks or in their relatively small territories (0.74 ± 0.44 ha, A. Russ, unpublished data) when not breeding, we assume this approximation to provide an appropriate estimate of the birds' exposure to the anthropogenic factors traffic noise and LAN.

Data of temperature and precipitation were obtained from the German Weather Service' online Weather Request and Distribution System (https://werdis.dwd.de) to calculate running means per decade of the daily mean temperature (*T*_10_ in °C) and running sums per decade of the daily precipitation (Prec_10_ in mm). These short‐term averages were expected to have a stronger influence on gonadal growth and regression than the highly variable daily mean temperature and sum of precipitation (Dawson 2008).

### Statistical analyses

Whether the two fecal sampling methods revealed equal distributions of the concentrations of T and E_1_S was tested by a Wilcoxon rank‐sum test. Because the test indicated no differences between the two sampling methods (T: *U* = 12078, *P* = 0.24, E_1_S: *U* = 12385, *P* = 0.08), it was not differentiated between the sampling methods in subsequent analyses. Collinearity between potential explanatory variables was assessed using Pearson correlation. All pairwise correlations were far from being collinear (¦*r*¦ ≪ 0.8) and a variance inflation factor below 2 for all explanatory variables further indicated independence of the predictor variables (Zuur et al. 2009).

Three separate analyses were conducted to evaluate the relationship between the avian endocrine physiology and potential predictor variables, one analysis for each hormone response. We used general additive mixed models (GAMMs) to identify the major environmental predictors and their interactions that determine individual levels of reproductive and stress hormones. GAMMs were fitted in the statistical software system R (version 3.1.2, R Development Core Team 2013) using the packages *mgcv* (Wood 2004) and *nlme* (Pinheiro et al. 2013). We log10(*x* + 1)‐transformed T and E_1_S concentrations and the *lamp density*, and centered and standardized all continuous explanatory variables to allow comparison of the size of estimated coefficients (Schielzeth 2010).

The initial models for the responses of T and E_1_S contained the *year* and the *sex* of the individual as fixed factors. Cubic regression spline smoothers were constructed for the continuous explanatory variables *day‐of‐year*,* sampling time*,* T*_10,_ and *lamp density*, each separately for males and females, while cubic regression spline smoothers of *Prec*_10_ were included for each year. Two‐way interactions between *day‐of‐year* and *lamp density* for each *sex* and *year*, as well as between *day‐of‐year* and *T*_10,_ for each *sex* were modeled as full tensor product smooths. To separate the influence of *sampling time* from the season, the *sampling time* was related to the length of day, starting with morning civil twilight (when the sun is 6° below the horizon) and ending with evening civil twilight when the sun is, again, 6° below the horizon.

In addition to the explanatory variables used in T and E_1_S models, the initial model of CORT included cubic regression spline smoothers of the elapsed time between capture and blood sampling (*handling time*), the *body condition*, the *car noise* and the *tramway noise*, the latter two separately for both males and females. The body condition was calculated as sex‐specific residuals from an ordinary least‐squares linear regression between body mass and tarsus length (Schulte‐Hostedde et al. 2005). As bird *handling times* ranged between 4 and 45 min (median = 12 min), which makes it difficult to differentiate between baseline CORT levels and a stress response, we restricted this analysis to samples for which the handling time did not exceed 10 min. This constraint reduced the sample size to *N* = 69 with 29 male and 40 female samples.

Because residuals showed heterogeneity between study years, different residual variances per *year* were included into the individual models of T, E_1_S, and CORT using the varIdent function (Zuur et al. 2009). To account for repeated sampling of birds, the individual was included as a random intercept in all models. The gonadal hormones T and E_1_S were further tested whether they correlate with CORT levels in individuals where both blood (taken within 10 min) and fecal samples were taken at the same day (*N* = 29). We built linear models with the log‐transformed T or E_1_S concentrations as response variable, included the linear and second‐order polynomial of day‐of‐year, as well as the sex, year, CORT, and T_10_ as fixed effects.

Model selection was based on Akaike's information criterion corrected for small sample sizes (AICc) (Wood 2006), an information‐theoretic approach (Burnham and Anderson 2002). All models with a ΔAIC_c_ < 4 of the respective best model were considered to receive competitive support and were included to calculate Akaike weights *ω*_i_ (Burnham and Anderson 2002).

## Results

### Exposure to light at night and traffic noise

Along the rural to urban gradient, the lamp density at sampling sites ranged from 0 to 46.3 lamps per hectare, with a median of 6.3, which corresponds to an illuminance of 0.17 lux (range 0.07–61.7 lux). Anthropogenic noise ranged from 35 to 63 dB(A) (mean ± standard deviation, 47.5 ± 6.5 dB(A)) and from 25 to 64 dB(A) (42.4 ± 9.7 dB(A)) for night‐time car and tramway traffic, respectively.

### T levels

**Figure 3:**
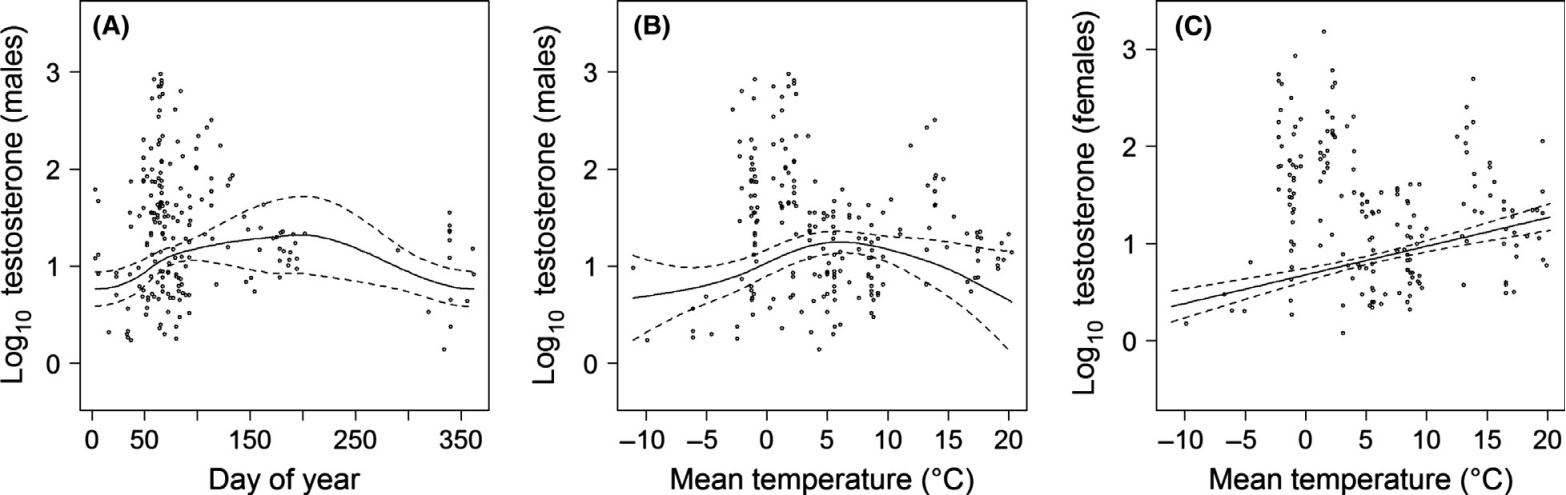
Smoothers related to day‐of‐year (A) and the mean temperature for males (B) and females (C) for the logarithmized testosterone concentration of blackbirds as predicted by the general additive mixed model (GAMM) with point wise 95 % confidence bands. The points indicate the distribution of the original data.

Over the whole study period, T levels ranged from 0.2 to 1505.2 ng/g dry matter with an overall median of 20.5 ng/g. The best model (edf = 14.5, *ω*_i_ = 0.69) indicated no effect of the lamp density on blackbird T concentrations but confirmed common patterns like significantly lower T levels for females (Table 1) as well as a clear annual cycle in male T levels with lowest concentrations in December and January and a steep increase in February (around *day‐of‐year* 50, Fig. 3). In addition to the annual cycle, male T concentrations were also significantly correlated to the mean temperature *T*_10_. T increased with rising mean temperatures, peaked around 7°C, and decreased above 10°C. In contrast, female T levels showed a continuous increase with rising mean temperatures (Fig. 3). There was a strong year effect which mirrored to some extent the unequal sampling effort, that is, in 2013, samples were almost exclusively collected during the breeding period when T concentrations are elevated, whereas in 2011, the nonbreeding season was overrepresented. Apart from this, the winter 2012/2013 was extraordinary mild but subzero temperatures occurred in February and March 2013 (month means −1.5 and −5.9°C below long‐term average, respectively). T levels were significantly negatively correlated to the precipitation in 2013 only (Table 1), and hence, Prec_10_ contributed to a better model fit, especially its interaction with year (Prec_10_ × year: *F* = 7.21, *P* < 0.001). No further model received statistical support.

**Table 1:** Model results for the variation in T, E_1_ S, and CORT. Models are general additive mixed models (GAMMs). The response variables T and E_1_S were log‐transformed. Reference level for year is 2011 and for sex is male

A) testosterone concentration				
Parameter	Level	Estimate ± SE	*t*	*P*
Intercept		0.89 ± 0.07	12.8	<0.001
year	2012	0.18 ± 0.06	2.88	0.004
	2013	1.12 ± 0.08	13.5	<0.001
Sex	Female	−0.23 ± 0.05	−4.63	<0.001
Prec_10_		0.06 ± 0.06	0.96	0.338
Prec_10_ × year	2012	0.04 ± 0.06	0.70	0.484
	2013	−0.16 ± 0.08	−2.07	0.040

Smooth terms	Level	edf	*F*	*P*
Day‐of‐year by sex	Male	2.94	2.00	<0.001
	Female	0.00	0.00	0.976
T_10_ by sex	Male	3.54	6.31	<0.001
	Female	1.00	42.1	<0.001

B) estrone sulfate concentration				
Parameter	Level	Estimate ± SE	*t*	*P*
Intercept		1.19 ± 0.09	12.8	<0.001
Year	2012	0.06 ± 0.09	0.63	0.527
	2013	0.03 ± 0.11	2.79	0.005
Sex	Female	0.05 ± 0.04	1.42	0.157
Lamp density		0.05 ± 0.03	1.61	0.108
T_10_		0.10 ± 0.04	2.48	0.014
Lamp density × sex	Female	−0.10 ± 0.04	−2.68	0.008

Smooth terms	Level	edf	*F*	*P*
Day‐of‐year		4.76	3.76	<0.001
Prec_10_ by year	2011	1.00	4.44	0.036
	2012	1.00	1.51	0.220
	2013	4.22	6.62	<0.001

C) corticosterone concentration				
Parameter	Level	Estimate ± SE	*t*	*P*
Intercept		35.34 ± 4.47	7.91	<0.001
Year	2012	−10.92 ± 3.37	−3.24	0.002
	2013	−23.7 ± 4.10	−5.80	<0.001
Sex	Female	−4.19 ± 1.91	−2.20	0.033
Body condition		−3.66 ± 0.95	−3.86	<0.001
Handling time		−8.00 ± 3.28	−2.44	0.019

Smooth terms	Level	edf	*F*	*P*
Day‐of‐year		4.43	6.06	<0.001
Lamp density by sex	Male	1.00	5.01	0.030
	Female	2.10	10.25	<0.001
T10 by sex	Male	2.27	55.91	<0.001
	Female	2.02	7.83	0.001
Tram noise by sex	Male	2.87	5.36	0.003
	Female	1.00	16.96	<0.001

### E_1_S levels

**Figure 4:**
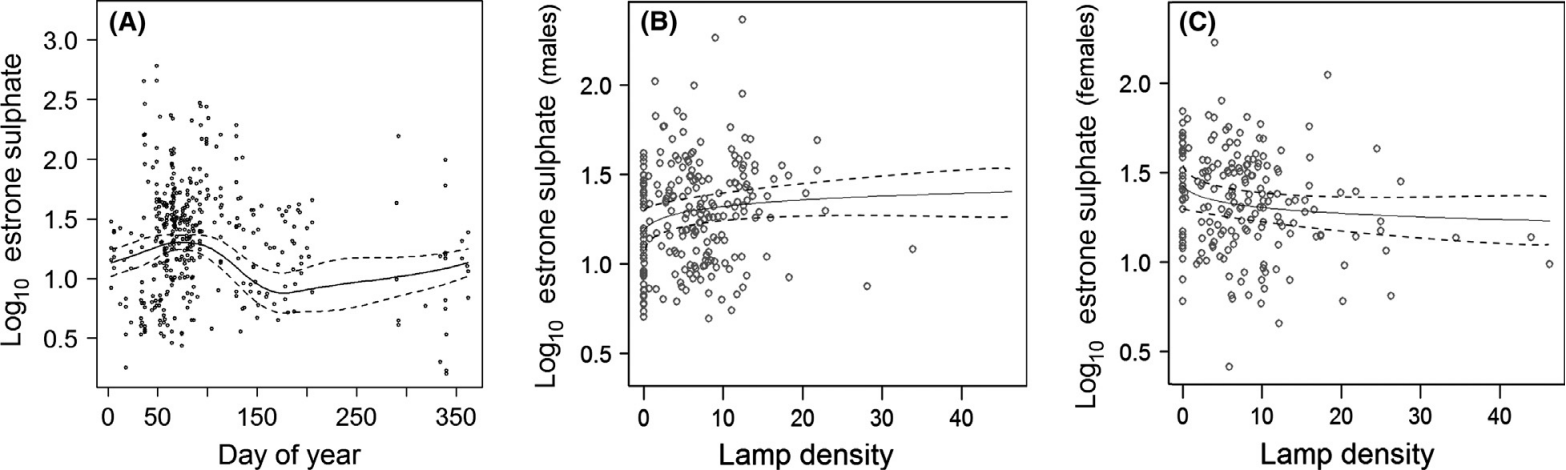
Smoother related to day‐of‐year (A) and lamp density for males (B) and females (C) for the logarithmized estrone sulfate concentration of blackbirds as predicted by the general additive mixed model (GAMM) with pointwise 95 % confidence bands. The points indicate the distribution of the original data.

The median concentration of E_1_S in fecal samples was 23.2 ng/g (range 0.6–604.5 ng/g) over the study period. The best ranked model (edf = 10.7, *ω*_i_ = 0.52) revealed equal E_1_S concentrations between the sexes. Also, E_1_S concentrations showed a significant seasonal pattern, they increased in February and March, declined thereafter until the end of May and showed intermediate values until the end of the year (Fig. 4). As in T concentrations, E_1_S levels differed between the years (*F* = 17.02, *P* < 0.001) with low levels in 2011 and 2012, whereas in 2013, significantly higher concentrations were measured, probably because most samples in 2013 were collected during the breeding season and the year effect is therefore partly confounded with seasonal variation. Mean temperature *T*_10_ had a weak but significant effect on E_1_S concentrations (Table 1), but the precipitation did not influence the E_1_S concentrations and was therefore excluded from the best model. Furthermore, the model indicated that the sexes differ in their response of E_1_S concentrations to the lamp density. A marginally significant negative effect is predicted for female E_1_S, indicating decreasing E_1_S with increasing lamp densities, while there is no effect on male E_1_S concentrations. Further candidate models with statistic support included smoothers of Prec_10_ per year (edf = 17.8, *ω*_i_ = 0.40) and excluded the temperature from the set of predictor variables (edf = 17.8, *ω*_i_ = 0.08).

### CORT levels

**Figure 5:**
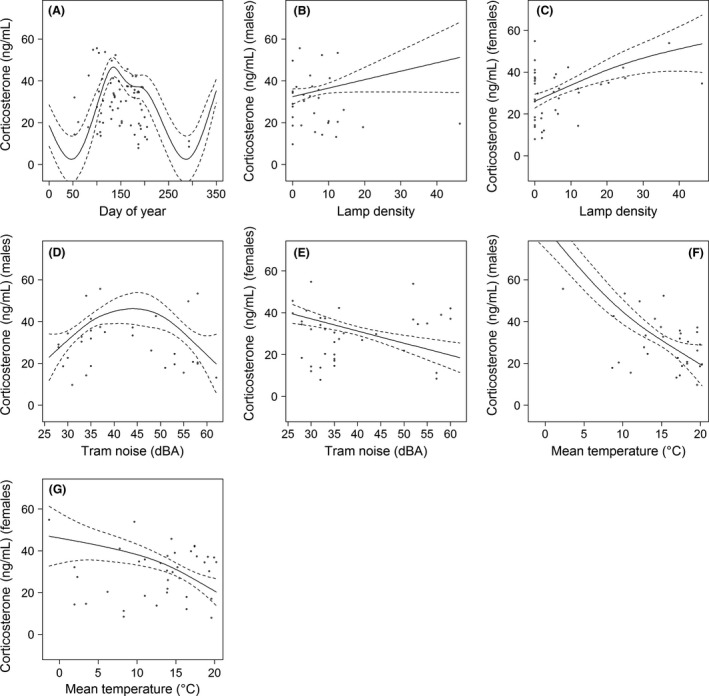
Smoother related to day‐of‐year (A), lamp density, tram noise, and mean temperature for plasma CORT of male (B, D, F) and female blackbirds (C, E, G) as predicted by the general additive mixed model (GAMM) with pointwise 95 % confidence bands. The points indicate the distribution of the original data.

Basal CORT levels during the study period averaged 29.5 ± 12.2 ng CORT/mL blood plasma (mean ± SD, range 7.9 – 55.6 ng/mL). Females had, on average, 12 % lower CORT levels than males, but both sexes showed equally decreasing CORT with higher body condition. The best model (edf = 21.7, *ω*_i_ = 1.00) indicated also clear differences between years (*F* = 17.66, *P* < 0.001) with the lowest CORT concentrations in 2013 (Table 1). During breeding, blackbirds exhibited elevated CORT levels, which peaked at the height of breeding in April and May and decreased toward the end of the reproductive period in July (Fig. 5A). Lowest CORT was predicted for autumn and a second CORT peak occurred in winter, but the base data are very limited for this time of the year. The precipitation had no effect while the CORT decreased with increasing mean temperature but the extent differed between the sexes (Table 1, Fig. 5F,G). Furthermore, there was no daytime (*sampling time*) effect but CORT levels decreased significantly with higher body condition of the birds and, paradoxically, also marginally significant with longer bird handling times (Table 1). The noise from *car traffic* did not affect the CORT concentrations and was excluded from the best model, while *tramway traffic* was negatively correlated with CORT levels in females and showed a unimodal pattern in male blackbirds (Fig. 5D,E). Additionally, both sexes showed a significant correlation with artificial LAN: the higher the lamp density the higher the CORT level (Fig. 5B,C). No other candidate CORT model received statistical support according to their AIC value.

The linear regression between the two reproductive hormones and CORT indicated a significant negative correlation between CORT and T levels (−0.017 ± 0.006, *F* = 15.84, *P* < 0.001), but not for E_1_S and CORT (*F* = 2.47, *P* = 0.129).

## Discussion

This study demonstrates that the reproductive physiology of wild European blackbirds is closely correlated with seasonal fluctuations in abiotic factors, whereas the stress physiology is also linked to individual condition, and anthropogenic factors.

Among the abiotic factors, photoperiod and increasing temperatures play a crucial role in determining the accurate time to start breeding in temperate birds (Dawson et al. 2001; Wingfield et al. 2003; Visser et al. 2009) which is often conveyed by elevated levels of reproductive hormones during spring (e. g. Smith et al. 1997). Here, the correlation of day‐of‐year with T and E_1_S levels confirm that the photoperiod provides a general cue of favorable conditions for breeding, whereas temperature and probably also precipitation allow fine‐tuning of the timing of reproduction due to year‐to‐year variations. A clear seasonal pattern was found for E_1_S levels but less definite in T levels. This might reason in T levels being less closely linked to photoperiod but stronger affected by year‐to‐year variations of abiotic or biotic factors, which is also indicated by the higher year effect and its interaction with the precipitation in T compared to E_1_S. However, in this study the predicted effect of year has to be handled with caution due to the unbalanced sampling effort in the three years of the study which impedes differentiating between effects of the year and the season. Regarding biotic factors, intraspecific aggression such as fighting territory intruders can elevate plasma T in males (reviewed in Wingfield et al. 1990; Parisot et al. 2005). Actually, these aggressive interactions might occur over almost the entire year, because a high percentage of the urban blackbirds refrain from migrating but remain in their breeding habitat, partly even defend territories and start breeding attempts in winter (Stephan 1999; Partecke and Gwinner 2007). Permanently elevated T, though, is disadvantageous for the individual as it may simultaneously suppress the immune function (Casto et al. 2001; Martin et al. 2008). Therefore, T may be converted into estrogens to mitigate the immunosuppression by reducing the concentration of circulating plasma T (reviewed in Hau 2007). Another possible but not mutually exclusive explanation lies in the extended breeding period of urban birds. Organisms with a relatively long breeding season may exhibit low to intermediate T levels over most of the reproductive season, which is only elevated during short phases of courtship and mating (Hau et al. 2000; Wingfield et al. 2001). We possibly missed those short events of drastically increased T by (1) catching blackbirds during the breeding season primarily while rearing offspring, hence, when peak T has already attenuated to provide parental care (reviewed in Hau 2007), and by (2) using fecal samples to determine T concentrations. In contrast to plasma T, fecal T levels show less variation and rarely reflect acute responses, but provide a cumulative measure over a prolonged period of time (Ninnes et al. 2010). The rate at which T is excreted in feces depends on the overall metabolism and defecation frequency of the organism (Palme 2005), but a reasonable estimate is 24–48 h (Tell 1997). Therefore, we do not find a daily pattern in T levels although there is evidence that it exists in plasma T (Kempenaers et al. 2008).

Nevertheless, despite applying the same methodology to obtain T and E_1_S concentrations, we find a strong correlation of E_1_S with the photoperiod. This strengthens our view that T levels are more susceptible to further environmental influences while E_1_S is more closely linked to the photoperiod. In line with this hypothesis, we find an effect of the lamp density for E_1_S but not for T levels. The marginal but significant effect of the lamp density on female E_1_S levels can be interpreted as perceived prolongation of the photoperiod. According to the annual cycle of reproduction and photorefractoriness (Dawson et al. 2001), the effect of a prolonged photoperiod might differ with the season and reproductive state of the bird. During late winter and early spring, while preparing for breeding, prolonged days should enhance the reproductive hormone levels, whereas a negative effect is expected during late breeding and molting in summer. However, our results indicate that higher lamp densities lower female E_1_S levels irrespective of the seasons (Table 1). This contradiction might erase from restricted accuracy of the chosen methods, particularly the light index included in our study. However, the corresponding night light intensities (mean 0.17 lux) fit well to those measured with light loggers attached to free‐ranging blackbirds (range 0–2.2 lux) and light treatment (0.3 lux) used in subsequent experiments to investigate the effects of LAN (Dominoni et al. 2013a).

Alternatively, the effect of the lamp density on E_1_S levels and the lack of a correlation in T levels can also be interpreted by the complex interrelationship of the endocrine system. An environmental cue which is perceived as a chronic stressor can trigger a stress response and affect the bird's reproductive physiology. CORT, the main avian glucocorticoid, mediates the endocrine stress response which downregulates the reproductive physiology at several levels of the HPG axis (Schoech et al. 2009). CORT decreases the expression of gonadotropin‐releasing hormone and the pituitary responsiveness to this neurohormone, which in turn suppresses the release of luteinizing hormone (LH), retards gonadal growth rates, and finally inhibits the release of sex steroids such as T and estradiol (E_2_) (rewied in Schoech et al. 2009; Goutte et al. 2010). Additionally, CORT acts via the gonadotropin‐inhibitory hormone (GnIH), which further downregulates the HPG axis (Schoech et al. 2009; Goutte et al. 2010). Although we did not find a significant correlation between E_1_S levels and CORT concentrations which might be due to the limited sample size and variations in environmental conditions, here, female CORT increased with higher lamp densities suggesting that LAN might act as such a chronic stressor and via the described pathways cause the observed decreasing female E_1_S levels. Likewise, in males, baseline CORT levels were positively correlated with the lamp density and the T levels in turn decreased with higher CORT levels. This interaction might have extenuated the seasonal variation in T levels although we did not find a direct correlation between T and the lamp density. Further support for this hypothesis comes, for example, from western scrub‐jays (*Aphelocoma californica*). Exposed to LAN, the scrub‐jays had lower LH, E_2_, and T levels than the dark controls (Schoech et al. 2013). Campo et al. (2007) reports that domestic chickens were significantly stressed under continuous light conditions, while diurnal rats exposed to dim LAN had elevated corticosterone levels, too, and showed an increased immune function (Fonken et al. 2012). However, why we do not find any effects in males and on T concentrations despite contrasting results in other studies (Partecke et al. 2005; Schoech et al. 2013) remains speculative but may be linked to the high variability seen in T levels.

Anthropogenic noise also possibly bears a stressful component as it was repeatedly shown to interfere with avian acoustic communication in urban environments (reviewed in Brumm and Slabbekoorn 2005). However, noise from car traffic did not affect the CORT in our analyses. Birds seem to be capable to avoid masking noise by changing temporal and acoustic traits of their song (e.g., Brumm 2004; Slabbekoorn and den Boer‐Visser 2006; Fuller et al. 2007) without perceiving noise as a stressor. Paradoxically, the noise from tramway traffic was predicted to have a mitigating effect on blackbird CORT (Fig. 5). However, this effect is difficult to interpret because tramway noise differs in its characteristics from traffic noise which is rather permanent. Tramway noise increases intermittently every time a tram passes by but between these noise peaks short fractions of time would allow acoustic communication between the birds. Since Rowan's first experiments on photoperiod and avian reproduction (Rowan 1925), numerous studies report a stimulating effect of experimentally increased day length or LAN on gonad recrudescence (e.g., Farner and Wilson 1957; Dominoni et al. 2013a). This seems to contrast with our results, but we do not exclude an advancing effect of LAN on the gonadal growth of the blackbirds in our study. Although we did not investigate the timing of gonad maturation, we found city blackbirds to start breeding earlier than their forest conspecifics (A. Russ, unpublished data) despite the moderate inhibition of E_1_S levels under high street lamp densities. Therefore, decreased E_1_S and possibly also T levels due to elevated LAN do not necessarily indicate suppressed reproduction. In contrast, reproduction or gonadal maturation can still be advanced, as observed in several studies, where overall T levels remained lower in light‐treated birds although they developed their gonads earlier than birds in dark controls (Partecke et al. 2005; Dominoni et al. 2013a; Zhang et al. 2014).

By now, the question persists why our results differ from those of laboratory experiments. An important aspect to consider in laboratory experiments is the comprehensive limitation to one or few environmental cues to assess the factor(s) of interest (Calisi and Bentley 2009). This restriction might also constrain the organism's plasticity because additional environmental cues certainly act in a complementary fashion. In the experiments of photoperiod and gonad recrudescence, birds are usually held under constant conditions of temperature and *ad libitum* food supply to measure the sole effect of photoperiod or LAN on gonad maturation. However, in nature, temperature and food availability underlie seasonal fluctuations like the photoperiod. In particular, the food availability plays an important role as supplemental factor in the decision to reproduce (reviewed in Ball and Ketterson 2008). Reproduction needs to be optimally timed so that food is available in sufficient amounts not only for adults, but also for the offspring during chick rearing (Dawson et al. 2001). Scarce food availability is perceived as stressful, and birds in an inferior body condition exhibit elevated CORT levels (Lanctot et al. 2003; Kitaysky et al. 2007), as our results also clearly indicate. Furthermore, males are more stressed than females probably due to intraspecific aggression, song production, or the maintenance of elevated T levels (Buchanan et al. 2001; Wingfield et al. 2001). As mentioned above, the release of CORT during stress responses shifts the trade‐off between reproduction and self‐maintenance toward the latter by redirecting energy toward physiological and behavioral adjustments that enhance the possibility to survive (Wingfield and Sapolsky 2003; Goutte et al. 2010). Hence, in an experimental setting, where food is provided *ad libitum*, the birds might overcome the stress triggered by LAN by ingesting additional food. In the wild, complying with higher energy demands might be a considerably selective pressure, especially in the context of additional stressors such as predation, intraspecific competition, or challenging weather conditions which are excluded in laboratory experiments. Hence, LAN might be perceived as longer or increasing photoperiod and, thus, stimulate gonadal recrudescence when further breeding requirements such as food availability, temperature, and nesting sites are not limiting.

In conclusion, our results suggest that LAN has the potential to interfere with the physiology of urban blackbirds. As we base these conclusions on observational findings, the causation remains to be tested because the found effects may be related to other influences which coincide with the presence of LAN. However, we suggest that LAN is perceived as a weak but chronic stressor that interacts with the HPG axis and leads to a reduced secretion of reproductive hormones. This interference might not have detrimental effects under optimal conditions or in species that appear to cope well with a life in urban areas such as the European blackbird, but may constitute an additional threat when environmental conditions deteriorate or in less stress‐tolerant species. As LAN rapidly increases by approximately 6 % per annum (Hölker et al. 2010a), the percentage of species that perceive LAN no further as a weak stressor but as a serious threat will certainly increase. The impacts of LAN are diverse, and we only slowly disentangle its multiple negative effects on ecology, biodiversity, and human health (Longcore and Rich 2004; Navara and Nelson 2007; Hölker et al. 2010b).

## Data Accessibility

The data sets supporting this article can be assessed at the Dryad Digital Repository as Russ et al. (2015) doi: 10.5061/dryad.kv32b (http://dx.doi.org/10.5061/dryad.kv32b).

## Conflict of Interest

None declared.
